# Time Perspective and Health Behaviors in Chronic Disease Patients: A Chain Mediation Model of Illness Perception via Temporal Self-Regulation Theory

**DOI:** 10.3390/bs15080996

**Published:** 2025-07-22

**Authors:** Xiaorong Lang, Sufang Huang, Yaru Xiao

**Affiliations:** 1 Department of Nursing, Tongji Hospital, Tongji Medical College, Huazhong University of Science and Technology, Wuhan 430030, China; lang5971996@163.com; 2 Department of Emergency, Tongji Hospital, Tongji Medical College, Huazhong University of Science and Technology, Wuhan 430030, China; huangsufang@tjh.tjmu.edu.cn

**Keywords:** time perspective, health behaviors, illness perception, motivation, temporal self-regulation theory

## Abstract

(1) The formation pathways and underlying mechanisms of health behaviors among young and middle-aged adults with chronic diseases under a temporal perspective remain underexplored. Based on Temporal Self-Regulation Theory and its subsequent extensions, this study aimed to investigate the time perspective among Chinese young and middle-aged adults with chronic diseases and analyzed the relationships between time perspective, illness perception, health behavior motivation, and health behaviors, thereby providing valuable empirical evidence for health behavior modification in this population. (2) This study was a cross-sectional survey conducted from March to April 2025. Structural Equation Modeling was employed to investigate the relationships among the variables. (3) This study enrolled a total of 391 participants with high levels of future and past-positive perspectives. Time perspective, directly related to health behaviors, namely past-negative, present-impulsive, and present-fatalistic perspectives, exerted negative associations with health behaviors, respectively, whereas future and past-positive perspectives showed positive associations with health behavior. Analysis of the mediating effect found that illness perception served as a mediator between the time perspective of past-negative, present-impulsive, and present-fatalistic perspectives, and health behaviors. And health behavior motivation acted as a mediator in the relationship between past-negative, present-impulsive, and past-positive perspectives, and health behaviors. Furthermore, illness perception and health behavior motivation formed a chain mediation pathway between time perspective and health behaviors. (4) Time perspective was associated with health behaviors, not only exerting directed effects but also manifesting a double-edged sword effect through illness perception and health behavior motivation. Subsequent interventions targeting health behaviors among young and middle-aged populations in China should incorporate considerations of the temporal perspective’s uniqueness and its intricate mechanisms of action on health behaviors.

## 1. Introduction

The rising prevalence of chronic diseases among China’s middle-aged and younger populations has evolved into a critical public health issue. It is reported that the prevalence of hypertension in China increased from 18.9% in 2002 to 29.6% in 2019, with the most significant rise observed among adults aged 18–39 years, where the prevalence nearly tripled (Zhang & Wu, 2025). And the prevalence of dyslipidemia and diabetes mellitus demonstrated marked increases across both 18–44 and 44–59 age groups, rising from 17.0%, 22.9%, 1.3%, and 4.3% in 2002 to 33.2%, 40.3%, 6.2%, and 16.1% in 2018, respectively (Peng et al., 2024).

Health behaviors have been widely recognized for their efficacy in reducing the risk of chronic diseases and mitigating adverse health outcomes associated with chronic conditions, such as a healthy diet that could significantly improve cardiometabolic profiles and aerobic exercise positively affecting blood lipid (Wood et al., 2023; Yiaslas et al., 2024). However, the impact of health behaviors on individual health is not instantaneous, particularly in the context of chronic disease management. Individual’s choice of health behaviors involves an intertemporal trade-off between immediate management costs and long-term health benefits (Ding et al., 2024). The fundamental motivation for individuals to adopt health-protective behaviors may stem from the pursuit of long-term benefits, which exhibit a temporal dimension (Hall & Fong, 2007). Therefore, considering the time perspective when exploring the factors and mechanisms behind the occurrence of health behaviors is one of the important contents of forming effective intervention programs.

Although prior studies have addressed the relationship between time perspective and health behaviors, research on the pathways through which time perspective influences health behaviors remains limited, particularly among middle-aged and young adults with chronic diseases in China. This study aims to explore the pathways by which time perspective operates on health behaviors and further elucidate the mediating roles mediated by illness perception and health behavior motivation, thereby providing valuable evidence for chronic disease management in middle-aged and young adult populations.

## 2. Theory and Hypothesis

### 2.1. Temporal Self-Regulation Theory

Interventions based on health behavior theories are recognized as effective approaches to improving individual health behaviors. Traditional health behavior intervention theories, such as the Theory of Planned Behavior (TPB), posit that intention is the decisive factor in behavior, establishing the “intention–behavior” link. This framework assumes that once an individual forms an intention to adopt a specific behavior, they will subsequently enact and maintain it. However, a significant gap persists between intention and actual behavior (Armitage & Conner, 2001). Most traditional models overlook the variability of behaviors, fail to account for connectedness beliefs, and lack a conceptualization of temporally distributed costs and benefits, which are critical for understanding health behavior performance over time (Hall & Fong, 2007).

Recognizing the critical role of temporal factors in individual health behaviors, Hall et al. proposed the Temporal Self-Regulation Theory (TST) (Hall & Fong, 2007). Similar to TPB, the TST posits that intention serves as the core determinant of behavior, with behavioral tendencies and control–execution resources moderating this relationship. And the TST further emphasizes that numerous antecedent factors influence behavior indirectly through their effects on intention. These influences can be captured through the temporal dimension of specific behaviors, reflecting an individual’s orientation toward proximal and distal outcomes of their actions. Time perspective is regarded as a unique and primary determinant of behavioral intention (Hall, 2013).

Given the model’s complexity and its multi-level analytical framework, fully testing the entire model is challenging, which has also been verified in subsequent related studies (Evans et al., 2017; Moran & Mullan, 2021). However, its components can be examined individually or in interrelated configurations (Hall, 2013). Aligning with the TST’s focus on bridging the intention–behavior gap, our study constructed a theoretical framework centered on the pathway “time perspective–intention–behavior”. Additionally, the TST suggests that an individual’s capacity for long-term goal-oriented behavior involves a complex interplay of cognitive factors (Hall & Fong, 2007). To operationalize this, we incorporate illness perception (a construct rooted in health behavior theories) as a novel variable in the model.

### 2.2. Time Perspective and Health Behaviors

Time perspective is defined as the extent to which an individual values and pays attention to the short-term and long-term consequences of a specific behavior, representing a cognitive–affective framework through which individuals automatically evaluate behavioral consequences across temporal dimensions (Hall & Fong, 2013). This implicit psychological operation organizes the continuum of lived experiences into distinct temporal zones, establishing systemic connections between sequential events to construct personal narratives and behavioral rationales, that is, endowing events with order, coherence, and meaning (Zimbardo & Boyd, 2015).

According to Zimbardo et al.’s conceptual framework, time perspective comprises five distinct dimensions: past-negative perspective reflects a generally pessimistic and aversive orientation toward past experiences, often characterized by rumination on adverse events; past-positive perspective embodies a warm, nostalgic attitude that actively reconstructs positive memories; present-fatalistic perspective denotes a helpless, deterministic outlook toward current circumstances; future perspective emphasizes proactive planning and goal-oriented behavior, prioritizing long-term rewards over immediate gratification; and present-hedonistic perspective represents a risk-tolerant, pleasure-focused approach to temporal experiences, valuing sensory enjoyment and spontaneity (Zimbardo & Boyd, 2015). Multiple studies have demonstrated significant associations between time perspective and health-related behaviors. For example, past-negative perspective might lead to alcohol dependence (H. Wang et al., 2022); past-positive perspective was found to be a protective factor against smoking (H. Wang et al., 2022); present-fatalistic perspective could reduce the frequency of exercise (Guthrie et al., 2014); a future-oriented time perspective was found to be associated with better dietary behaviors, and individuals with a stronger view of future time were more likely to adhere to healthy behaviors (Jin et al., 2023; Sun et al., 2023); and present-hedonistic perspective could increase the risk of material abuse (Lee & Liao, 2022). However, there are differences among various research results. The strongest prediction of the time perspective of health behavior has not been uniformly determined yet. And time perspective and health behavior are full of complexity and uncertainty. The mechanisms by which time perspective affects health behavior remain unclear.

### 2.3. Mediating Roles of Illness Perception and Health Behavior Motivation

Illness perception refers to the process by which individuals cognitively evaluate their health status based on prior knowledge and experiences (Guo et al., 2025). It constitutes a subjective mental representation encompassing personal cognitive appraisals, emotional responses, and comprehension of the disease and its potential consequences, which can reflect the level of threat posed by the disease perceived by the individual (C. He et al., 2025). The predictive role of illness perception in health behaviors has been empirically validated. Studies demonstrate that patients’ illness perception directly influences adherence to healthy lifestyles (Al-Faraj et al., 2025). For instance, enhanced illness perception was associated with stronger compliance with low-salt diets and abstaining from alcohol, whereas poorer illness perception might lead to reduced engagement in physical activity (de Vries et al., 2025; Larki et al., 2018). Although, to the best of our knowledge, there is currently no direct research linking time perspective with illness perceptions. According to the Common Sense Model (CSM) of illness representations, one of the core dimensions of illness perceptions is timeline, which in turn influences health-related behaviors (O’Donovan et al., 2020). Therefore, we supposed that illness perceptions play a significant mediating role between time perspective and health behaviors.

Behavioral intention, as the direct driving force behind behavior occurrence, serves as an intermediate variable between time perspective and health behavior, a role explicitly demonstrated in the TST. As emphasized by the Health Belief Model, an individual’s perceived threat of disease is a critical determinant in the formation of behavioral intentions. When individuals perceive a heightened disease threat, their behavioral intentions are amplified, manifesting in enhanced health-promoting practices. Consequently, illness perception and health behavior motivation may act as serial mediators in the relationship between time perspective and health behavior.

**Figure 1:**
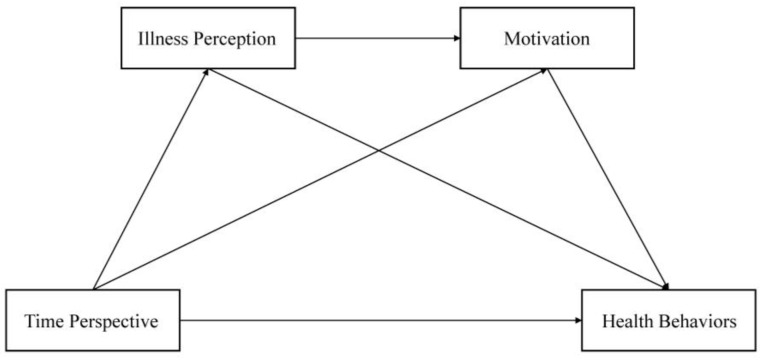
The hypothetical theoretical model.

Based on the TST and a review of the literature, we propose the following hypothesis, and the hypothetical model is shown in Figure 1:

**Hypothesis 1 (H1):**  *Time perspective, illness perception, health behavior motivation, and health behaviors were closely interrelated among middle-aged and young adults with chronic conditions.*

**Hypothesis 2 (H2):**  *Time perspective, illness perception, and health behavior motivation all exerted direct associations with health behaviors.*

**Hypothesis 3 (H3):**  *Both illness perception and health behavior motivation served as mediating variables in the pathway through time perspective and health behaviors.*

**Hypothesis 4 (H4):**  *Illness perception and health behavior motivation formed a chain mediation model in the temporal pathway linking time perspective to health behaviors.*

## 3. Materials and Methods

### 3.1. Participants and Procedures

This study consisted of a cross-sectional study that was conducted from March to April 2025. The data was collected via Credamo during the period from March to April 2025. Credamo is a professional research platform that boasts over 3 million registered participants, spanning all provinces and regions in China (Credamo, 2025). It has established collaborations with numerous scientific research institutions, encompassing a wide range of disciplines within the academic fields of management, psychology, medicine, sociology, tourism, and hotel management (Credamo, 2025). Credamo has gained recognition in the academic community, with multiple behavioral studies conducted utilizing this platform having been published in reputable journals (J. He et al., 2024; Lau et al., 2024; J. M. Wang & Li, 2022).

We initially randomly recruited participants through a brief questionnaire encompassing demographic and health status surveys. Following a screening process, eligible candidates meeting the study criteria were enrolled in the sample pool. Within two weeks, the full questionnaire was distributed by targeted delivery to the selected participants via a preconfigured system. The inclusion criteria required participants to be aged 18–59 years and diagnosed with at least one chronic disease (e.g., cardiovascular disorders, diabetes, chronic respiratory conditions, or chronic diseases of the digestive system). Fraudulent and careless responses were excluded. Monetary compensation was provided at two stages: RMB 0.5 during recruitment and RMB 5 upon completing the formal survey, serving as financial incentives to enhance participation rates.

### 3.2. Sample Size

Firstly, the sample size was determined through an a priori power analysis using G*Power 3.1. For the primary objective (investigating the relationships among variables), we set α = 0.05 (two-tailed), power = 0.80, and an effect size of r = 0.30. This yielded a minimum requirement of 82 participants. However, this study aimed to construct a Structural Equation Modeling (SEM) to explore the paths among variables, yet a sample size below 200 may lead to insufficient statistical power and compromise the reliability of the research findings (Deng et al., 2018). Furthermore, this study employed a cross-sectional design; the calculation formula (N = $\frac{Z_{\alpha /2}^{2}\;\times \;p\;\times \;(1-p)}{d^{2}}$, $z_{\alpha /2}^{2}$ = 1.96, d = 5%), which is based on prevalence rate and is applicable to cross-sectional studies, was reused to compute the required sample size. According to prior epidemiological studies, the prevalence of chronic diseases among young and middle-aged adults in China has been reported to range from 6.2% to 37.4% (Y. Chen et al., 2023; Peng et al., 2024; Zhang et al., 2023; Zhang & Wu, 2025). Therefore, according to the sample size requirements for SEM and the aforementioned calculation formula, the valid samples included in this study were determined to be at least 360 cases.

### 3.3. General Demographic Data

The general demographic information of the participants included gender, age, place of residence, marital status, education level, employment status, and monthly income.

### 3.4. Assessment Instruments

#### 3.4.1. Zimbardo Time Perspective Inventory

The Zimbardo Time Perspective Inventory (ZTPI) was initially developed by Zimbardo et al. to assess an individual’s orientation toward time (Zimbardo & Boyd, 2015). Through cross-cultural adaptation by international scholars, this scale has been translated into multiple language versions for research applications. The current study employed a Chinese version of ZTPI (ZTPI-C) that has been culturally adapted to align more closely with Chinese sociocultural contexts. ZTPI-C, revised by Li et al. (2023), consists of 25 items and measures five dimensions: past-negative, present-impulsive, future, past-positive, and present-fatalistic perspectives. It employs a 5-point Likert-type scoring system, with responses ranging from 1 (extremely uncharacteristic of me) to 5 (extremely characteristic of me). This differed slightly from the original scale, as the dimensions revised after cultural adaptation no longer include items reflecting hedonism. Consequently, “present-hedonistic” has been renamed to “present impulsive”, which was regarded as a characteristic of impulsiveness, carelessness, and disregard for consequences (Li et al., 2023). The revised version demonstrated acceptable reliability in the large sample survey, with Cronbach’s alpha coefficients for the dimensions being 0.75, 0.69, 0.67, 0.73, and 0.62, respectively (Li et al., 2023). The Cronbach’s alpha coefficients in this study were 0.837, 0.800, 0.722, 0.765, and 0.677, respectively. Considering that the subscale of present fatalism had a Cronbach’s alpha coefficient below 0.7 during its initial development and contained only three items (a small number of items can lead to a lower alpha value, rather than indicating insufficient questionnaire reliability), the value of 0.677 obtained in this study was deemed acceptable (Streiner, 2003).

#### 3.4.2. Brief Illness Perception Questionnaire

The Brief Illness Perception Questionnaire (BIPQ) was developed by Broadbent et al. to assess the perceived threat of illness, consisting of eight Likert-scale items and one open-ended question investigating causal attribution (Broadbent et al., 2006). This instrument assesses three core dimensions: cognitive illness representations, emotional representations, and illness comprehensibility. Except for the causal attribution question, all items are rated using a 10-point Likert scale. Higher total scores reflect stronger illness perceptions. Although the Cronbach’s alpha coefficients of the questionnaire varied across studies with different themes, the surveys conducted in Chinese populations demonstrated good reliability (L. Chen et al., 2025; Guo et al., 2025). The Cronbach’s alpha coefficient was 0.640 in this study, which was consistent with previous survey findings in young people with chronic diseases (Liu et al., 2024).

#### 3.4.3. Treatment Self-Regulation Questionnaire

The Treatment Self-Regulation Questionnaire (TSRQ) was utilized to assess participants’ motivation (Csdt, 2025). The utilization of this questionnaire as a tool for assessing motivation in health behaviors has been acknowledged (De La Cruz et al., 2021; De Los et al., 2023). TSRQ comprises 15 items across four dimensions: autonomous motivation, introjected regulation, external regulation, and amotivation. Each item is rated on a 7-point Likert scale ranging from “1 = not at all true” to “7 = completely true”, with higher total scores indicating stronger motivation. The Chinese version of this questionnaire has been widely employed in research and has shown good reliability and validity across studies (H. Tang et al., 2025; Tao et al., 2024). The Cronbach’s alpha coefficient was 0.711 in this study.

#### 3.4.4. Health Promoting Lifestyle Profile—II

The Chinese version short form of the Health Promoting Lifestyle Profile—II (HPLP-IICR) was used to assess health behaviors. HPLP-IICR was revised by Teng et al., including 30 items with five dimensions: spiritual growth, physical activity, health management, nutrition, and health responsibility (Teng et al., 2010). All items were scored using a four-point Likert scale (1 = never, 2 = sometimes, 3 = often, and 4 = routinely), with higher total scores indicating better health behaviors. The Cronbach’s alpha coefficient of the questionnaire was 0.90, indicating good reliability. The Cronbach’s alpha coefficient was 0.891 in this study.

### 3.5. Identifying Fraudulent and Careless Responses

Although online receipt collection provided an efficient and convenient means for research, concerns have been raised regarding participants’ fraudulent and careless responses. Therefore, following recommendations from prior studies, we implemented preventive measures to mitigate such issues. Prior to commencing the formal questionnaire, participants were explicitly informed that monetary compensation would be provided exclusively upon acceptance of their submitted responses by the research team. And each question is preceded by a standardized prompt: “Please select answers based on your genuine perspectives. Submitted responses will undergo rigorous logical validation checks by the research team. Non-compliant entries will be excluded from analysis.” Additionally, to mitigate automated or inattentive responses, we embedded strategically designed attention-check items (e.g., “The sun appears at night”) (Ward & Meade, 2023). The concluding section incorporates self-report items assessing participant meta-cognition (e.g., “I thoroughly reviewed each questionnaire item”, “I provided dishonest responses to certain questions”) (Ward & Meade, 2023). After data retrieval, submissions with temporal anomalies were excluded based on individual circadian rhythms due to potential automated bot activity (i.e., responses submitted before 7:00 or after 24:00) (Comachio et al., 2024). Additionally, entries with IP addresses geographically inconsistent with self-reported provincial locations were disregarded, given that IP geolocation reflects participants’ approximate geographical scope during questionnaire completion (Comachio et al., 2024). Furthermore, the full questionnaire mandated participants to re-report their chronic disease status, which was cross-checked with the initial reports to minimize misrepresentation. Finally, the responses provided by participants were thoroughly examined, and any responses exhibiting clear inconsistencies were excluded from the analysis, as they were deemed not to have been completed with sufficient care.

### 3.6. Statistical Analysis

Descriptive analyses were conducted using IBM SPSS Statistics version 26.0. The description of categorical variables is presented in terms of their counts and proportions. Central tendency and variability of continuous variables were summarized using means (M) and standard deviations (SDs). The online tool developed by Tang et al. was used to generate a correlation matrix plot between variables, employing Pearson correlation analysis with a two-tailed test (D. Tang et al., 2023). To illustrate the relative relationships among the dimensions of time perspective while accounting for variations in the number of test items across dimensions, we calculated per-item mean scores to derive standardized dimension scores, which were then visualized using a radar chart. Non-parametric tests were employed to assess the differences between two independent groups. To mitigate risks of common method variance in self-reported measures, Harman’s single-factor test was performed prior to model testing. We used PROCESS Model 6, which tests serial mediation through two mediators (illness perception → health behavior motivation → health behaviors) while controlling for direct effects of time perspective dimensions on both mediators and the outcome variable, and 5000 bias-corrected bootstrap resamples were employed to enhance precision in estimating indirect effects that preferred for its robustness to non-normal sampling distributions. Standardized path coefficients were reported, and general demographic variables were included as covariates to control for extraneous variance. Mediation effects were considered statistically significant if the 95% bias-corrected confidence interval (CI) excluded zero.

## 4. Results

### 4.1. Characteristics of Participants

We initially recruited 600 participants. During the formal survey phase, 564 responded, among whom 11 were excluded due to abnormal response timing, 5 due to discrepancies in chronic disease reports compared to the recruitment phase, and 157 due to location inconsistency with their IP addresses, yielding a final sample of 391 checked participants. Notably, female participants predominated, accounting for 62.4%. The population demonstrated a relatively young profile, with a mean age of 33.12 ± 8.77 years. The majority of participants held a bachelor’s degree or higher (84.9%). Participants in this study were in-service staff (84.9%). Among the self-reported chronic conditions, chronic digestive system diseases were the most prevalent (44.5%), followed by endocrine and metabolic disorders (43.0%) and cardiovascular diseases (29.9%). Detailed characteristics of the participants are summarized in Table 1.

### 4.2. Common Method Bias Test

When all items from the ZTPI-C, BIPQ, TSRQ, and HPLP-IICR were included in Harman’s single-factor test and subjected to exploratory factor analysis, 20 common factors were extracted. The first common factor accounted for 16.70% of the total variance explained, which is below the critical threshold of 40%, indicating no severe common method bias in this study (Podsakoff et al., 2003).

### 4.3. Description of Variable Distributions and Correlations

**Figure 2:**
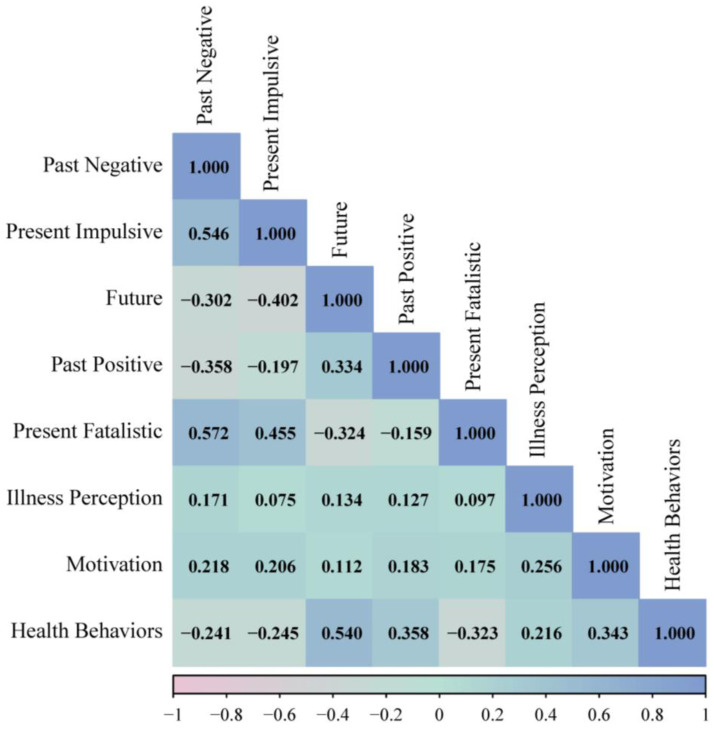
The heat map illustrating the correlation among variables.

**Figure 3:**
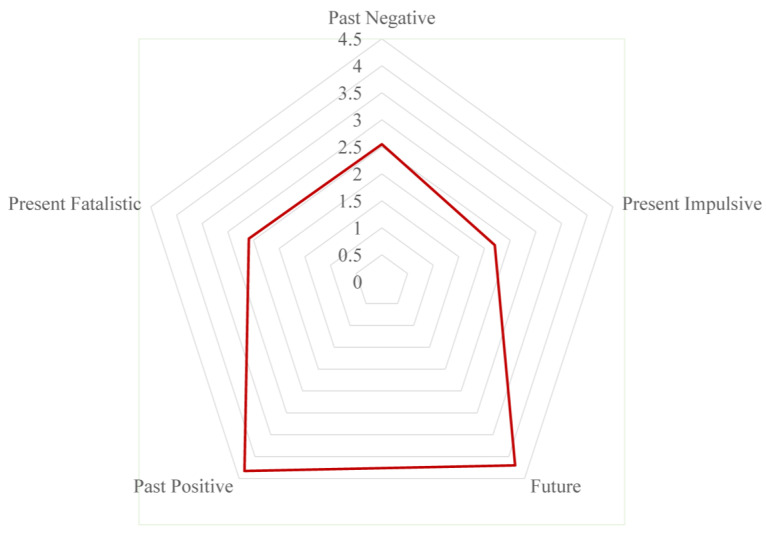
The radar chart of time perspective.

**Figure 4:**
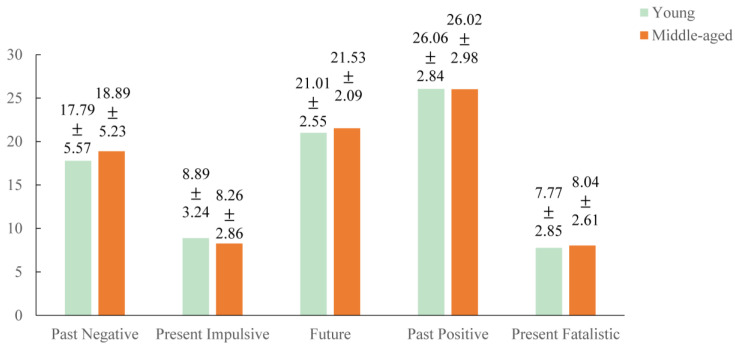
Scores of time perspective in the youth and middle-aged groups.

**Figure 5:**
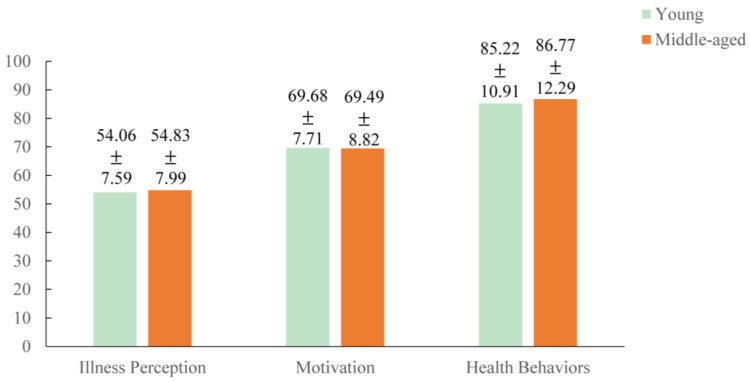
Scores of illness perception, health behavior motivation, and health behaviors in the youth and middle-aged groups.

The mean scores and standard deviations of multidimensional time perspectives, illness perception, health behavior motivation, and health behaviors are presented in Table 2. As shown in Figure 2, significant intercorrelations existed among time perspective, illness perception, health behavior motivation, and health behaviors, and the intensity of correlation reached at least a medium level (r > 0.1) according to Cohen’s guidelines (Gaskin & Happell, 2013), supporting H1. As demonstrated by the results of the radar chart in Figure 3, participants in this study were characterized by high levels of future and past-positive perspectives. In addition, non-parametric test comparisons showed that there were no significant differences (*p* > 0.05) in time perspective, illness perception, health behavior motivation, and health behaviors between the young and middle-aged groups. And scores for those variables in both the youth and middle-aged groups are presented in Figure 4 and Figure 5.

### 4.4. Model Verification and Mediating Effect Test

The fit indices of structural equation models constructed based on the five dimensions of time perspective were all $\chi^{2}$/df = 0, CFI = 1, TLI = 1, RMSEA = 0, and SRMR = 0, demonstrating acceptable data fit, since the standard criteria for evaluating model fit indicate that CFI and TLI > 0.90, RMSEA < 0.08, and SRMR < 0.08 signify acceptable fit (Xia & Yang, 2019).

**Figure 6:**
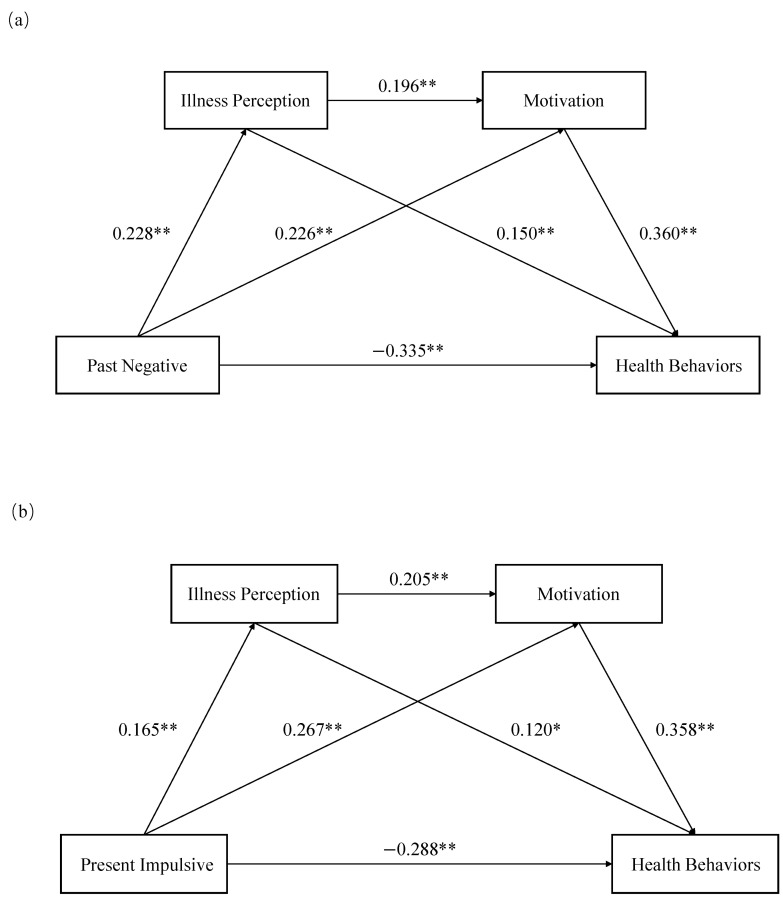

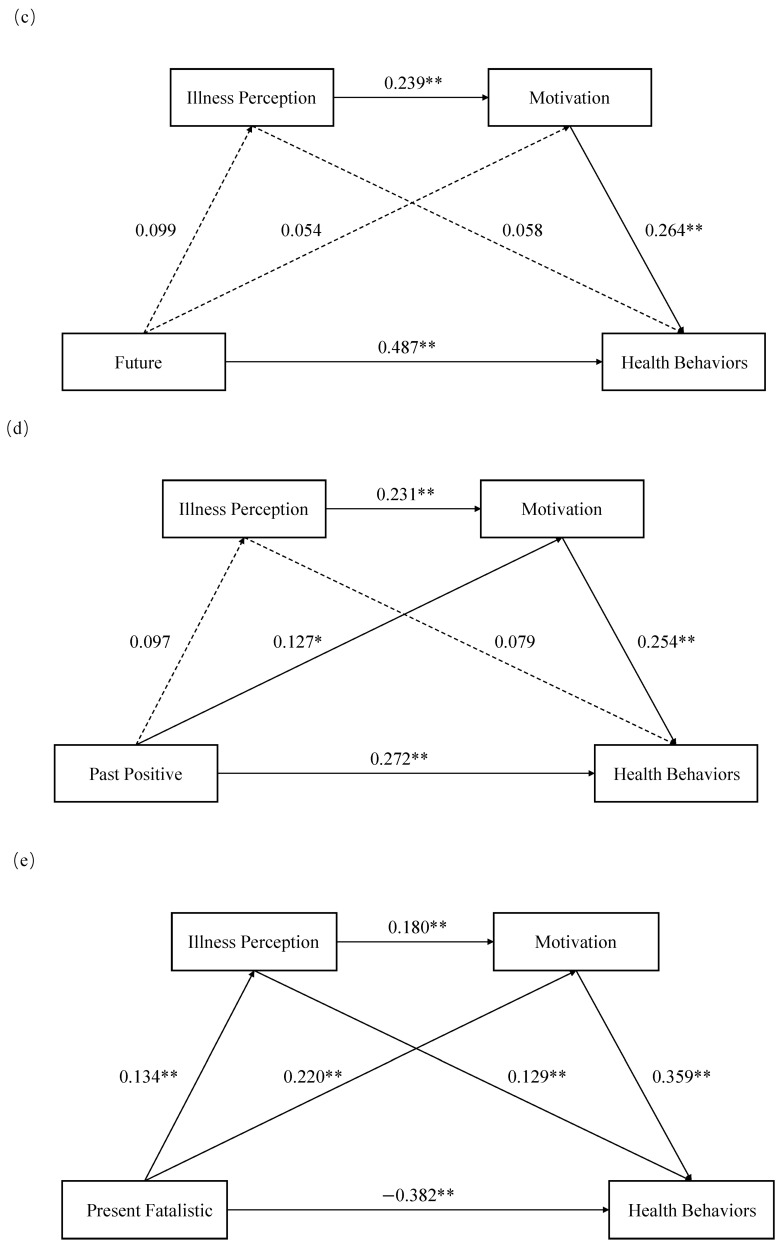
The verified chain mediation model: (**a**) Path relationship between past-negative perspective and health behaviors. (**b**) Path relationship between present-impulsive perspective and health behaviors. (**c**) Path relationship between future perspective and health behaviors. (**d**) Path relationship between past positive perspective and health behaviors. (**e**) Path relationship between present-fatalistic perspective and health behaviors. The path coefficients presented in the figure are all standardized estimates. * and ** indicate statistical significance at *p* < 0.05 and *p* < 0.01, respectively. Effect sizes of path coefficients: (1) small: 0.1 ≤ absolute value; medium: 0.3; and large: absolute value ≥ 0.5. The solid arrow and dotted arrow correspond to *p* < 0.05 and *p* > 0.05, respectively.

This study validated the proposed hypotheses through model construction. The analysis revealed that the pathway between time perspective and health behaviors was significant, and time perspective was directly related to health behaviors. Specifically, past-negative (effect size: −0.670, 95% CI: −0.858 to −0.481), present-impulsive (effect size: −0.997, 95% CI: −1.337 to −0.656), and present-fatalistic (effect size: −1.503, 95% CI: −1.846 to −1.160) perspectives exerted negative associations with health behaviors, respectively, whereas future (effect size: 2.155, 95% CI: 1.789 to 2.522) and past-positive (effect size: 1.057, 95% CI: 0.708 to 1.406) perspectives showed positive associations with health behaviors, thereby confirming H2. However, the mediating role of illness perception was only observed in past-negative (effect size: 0.068, 95% CI: 0.024 to 0.130), present-impulsive (effect size: 0.069, 95% CI: 0.011 to 0.154), and present-fatalistic (effect size: 0.068, 95% CI: 0.008 to 0.158) perspectives. And the mediating effect of health behavior motivation manifested on past-negative (effect size: 0.162, 95% CI: 0.077 to 0.256), present-impulsive (effect size: 0.330, 95% CI: 0.176 to 0.499), past-positive (effect size: 0.126, 95% CI: 0.009 to 0.259), and present-fatalistic (effect size: 0.254, 95% CI: 0.101 to 0.426) perspectives, providing partial support for H3. Furthermore, the serial mediation effects involving both illness perceptions and health behavior motivation were statistically significant within the time perspective framework, which validated H4. The structural path model and effect size tests are detailed in Figure 6 and Table 3 and Table 4, respectively.

## 5. Discussion

Our study explored the time perspective of Chinses young and middle-aged chronic disease patients and constructed a chain mediation model to examine the intricate interplay and underlying mechanisms among time perspective, illness perception, health behavior motivation, and health behaviors, demonstrating that illness perception and health behavior motivation mediate the relationship between time perspective and health behaviors, which reaffirmed the validity of the TST model and provided critical empirical evidence elucidating the interconnection between temporal cognition and disease-related cognitive schemas.

The participants in our study exhibited relatively high time perspective scores in the future (standardized dimension score: 4.2) and past-positive (standardized dimension score: 4.33) perspectives. This discrepancy appears reasonable and is supported by theoretical foundations, given that previous research demonstrated that younger individuals exhibited a stronger future time perspective, with future time horizon and age forming an inverted U-shaped relationship (Allemand et al., 2025; Kooij et al., 2018). As age increases, the future time perspective tends to strengthen until middle age, after which it gradually diminishes. In our study, the participants were predominantly middle-aged and young adults, which elucidated the differences in temporal perspective observed and highlighted variations in the dominant dimension of temporal perspective across specific age groups. Furthermore, compared to Western populations, Chinese individuals conceptualize time with a past-in-front and future-at-back orientation (Gu et al., 2019). And across cross-cultural contexts, a universal tendency to prioritize positive events persists, which provides informational scaffolding for life goals (Y. Wang & Singer, 2020; Zaragoza et al., 2015). This shared cognitive pattern may explain why the past-positive time perspective emerged as the most salient dimension in this study.

Time perspective serves as a robust predictor of healthy behaviors. Our research findings indicated that positive time perspectives fostered health-promoting behaviors, whereas negative time perspectives were associated with detrimental health outcomes. Specifically, future and past-positive orientations were identified as significant positive predictors of health behaviors, while past-negative, present-impulsive, and present-fatalistic orientations exhibited negative predictive effects, and the future time perspective emerged as the strongest predictor of health behaviors (standardized path coefficient: 0.487), a result consistent with prior studies (Henson et al., 2006; Kooij et al., 2018; Villaron et al., 2017).

Our study revealed that past-negative (standardized path coefficient: 0.228), present-fatalistic (standardized path coefficient: 0.134), and present-impulsive (standardized path coefficient: 0.165) temporal orientations significantly predicted heightened illness perception, which corroborated that time perspective exhibits a close relationship with illness perception, especially negative time perspective. Considering the CSM points out that illness perception involves both cognitive and emotional pathways, this result was attributed to illness perception originating from past experiences in a negative time perspective, and the exacerbation of illness manifestations by negative affectivity (O’Donovan et al., 2020). Interestingly, not all temporal dimensions demonstrated predictive validity. Future and past-positive perspectives showed non-significant associations with illness perception. This pattern was further reinforced in mediation analyses, indicating that illness perception does not mediate the effects of future and past-positive orientations on health behaviors. We attributed this phenomenon to the temporal particularity of illness perception. Illness perception constitutes an immediate evaluation based on prior experiences of current symptoms, consequences, and controllability rather than a focus on long-term consequences (Guo et al., 2025; Zimbardo & Boyd, 2015), which created a temporal framework mismatch between future and illness perception. Additionally, future time perspective and past-positive orientation, which encompass optimistic emotions, might reduce disease alertness. Moreover, the predominant temporal dimensions among participants in this study—past-positive and future perspectives—could further exacerbate inconsistency with current disease appraisal, obscuring the mediating effect of illness perception.

The core tenet of the TST, namely the robustness of the “time perspective–intention–behavior” linkage, was reaffirmed and expanded through empirical validation. Our findings demonstrated that, except for the future-oriented temporal perspective, all other dimensions (past and present) enhanced health behaviors by modulating health behavior motivation. According to the TST, behavioral intentions are formed based on expectancy–value components (Hall & Fong, 2007). Individuals with a past-positive perspective tended to perceive health behaviors as more rewarding due to their positive historical experiences, thereby enhancing their valuation of health-related outcomes and strengthening motivation to engage in such behaviors. Notably, negative temporal perspectives (e.g., past-negative, present-fatalistic, and present-impulsive perspectives) also amplified health behavior motivation, revealing a counterintuitive pattern that contrasted with prior research from other countries (Braitman & Henson, 2015; Chew et al., 2019). Synthesizing the cultural foundation of “being vigilant in times of safety” within the unique Chinese cultural context and Hall’s assertion regarding the uncertainty and emotional volatility inherent in the TST, negative temporal perspectives might act as a “double-edged sword”: crisis awareness was embedded into individual health management, linking past negative health experiences with future health outcomes, thereby being imbued with time tension. While reducing future-oriented optimism, individuals amplified their recognition of potential adverse health outcomes through prospective affective appraisals, consequently enhancing health behavior motivation and improving health behaviors, rather than exerting detrimental effects on health behaviors (Hall & Fong, 2007; Jiang & Yu, 2025). For instance, individuals with a present-fatalistic perspective might overestimate the inevitability of negative health trajectories, thereby paradoxically heightening their motivation to adopt preventive behaviors as a compensatory mechanism, which provides a new perspective for the intervention of health behaviors.

Another novel finding in our study was that illness perception and health behavior motivation act as sequential mediating variables between time perspective and health behaviors. While acknowledging the suboptimal reliability of the BIPQ, which necessitated cautious interpretation of results, it was noteworthy that time perspective consistently demonstrated significant direct and total effects on health behaviors. Even if a single mediating pathway is not statistically significant, further examination of chain mediators remains meaningful and insightful (Zhao et al., 2010). Moreover, a core hypothesis of this research was to test the validity of the chain mediation model. The results demonstrated that when illness perception and health behavior motivation were examined as a sequential chain, the hypothesized pathway became statistically robust. As previously discussed, the unique manifestations of time perspective among Chinese populations—particularly the weaker effects of future-positive and past-positive orientations on illness perception and health behavior motivation—highlighted the cumulative nature of mediation effects across variables. This underscored the cumulative nature of mediating effects among variables—a synergistic interaction that ultimately enabled the integrated chain mediation pathway to function as a whole, thereby confirming the scientific validity of our extended theoretical model.

By integrating illness perception into the TST framework, we established a chain mediation mechanism that aligns with real-world behavioral patterns. Unlike theories that focus solely on the motivation–behavior linkage or incorporate a temporal dimension but omit the motivational component (e.g., TPB, CSM), our findings provide a crucial theoretical framework for health behavior interventions of people with chronic disease, elucidating the complex dynamics of health cognition under distinct temporal perspectives, which underscores the necessity of integrating temporal cognition (temporal perspective), psychological representations (illness perception), and motivational drivers (health behavior motivation) to design holistic health behavior change strategies. For instance, reshaping the temporal perspectives to strengthen the alignment between time perception and illness awareness can enhance disease consciousness (e.g., employing Acceptance and Commitment Therapy to reduce past rumination and focus on present objective reality), strengthen future orientation (e.g., implementing future self-continuity training to recognize long-term adverse outcomes of the disease through a future-oriented lens), or leverage cultural contexts to amplify disease severity perceptions through either negative or positive temporal perspectives. Moreover, such an approach can fortify motivation by further providing external support (e.g., leveraging group dynamics and professional guidance) or activating intrinsic drives (e.g., linking health behaviors to profound life goals, translating abstract risks into concrete threat perceptions, and implementing a dual-drive incentive mechanism combining positive/negative reinforcement) to enhance motivation for health behaviors, thus promoting and maintaining behavioral change. However, the feasibility of such an approach in practice requires ongoing investigation and refinement; the formulation of intervention strategies must take into account the characteristics and preferences of the intervention targets, as psychology and behavior remain complex issues.

Although our research elucidated the trajectory of healthy behavior formation from a temporal perspective and offered valuable insights for exploring healthy behaviors, certain limitations remained. Firstly, the cross-sectional design cannot determine whether variables occur simultaneously or exhibit reciprocal causation; hypothetical models are established solely based on theoretical assumptions. This inherently limits causal inference in mediator models and captures data only at specific time points. Given that time perspective constitutes a long-term, dynamically evolving construct influenced by individual, social, and institutional factors, our methodological approach failed to account for its inherent temporal fluidity. Furthermore, mutually reinforcing relationships may potentially exist among time perspective, illness perception, motivation for health behaviors, and health behaviors themselves. Future research should adopt multi-wave designs to examine the dynamic interplay between fluctuations in time perspective and the formation of long-term health behaviors. Secondly, while participants were recruited nationwide through online platforms, the sampling strategy may not precisely reflect population demographics. Web-based recruitment, though cost-effective, requires participants to have internet access and technological literacy, risks overrepresenting tech-savvy subgroups and underrepresenting populations with limited internet access (e.g., rural communities). This selection bias could compromise the generalizability of findings. To enhance external validity, future research should integrate stratified sampling with offline recruitment channels or utilize nationally representative surveys anchored in probabilistic frameworks. Additionally, the reliability of some measures, particularly the present fatalistic subscale and Brief Illness Perception Questionnaire, was below optimal thresholds (a Cronbach’s alpha coefficient is generally considered to be greater than 0.7), which may lead to compression of the observed effect size, attenuation of direct effects, and increased sensitivity to confounding bias, thereby compromising the robustness of the results.

## 6. Conclusions

Our research findings indicated that time perspective, illness perception, health behavior motivation, and health behaviors exhibited intricate and multifaceted interconnections. Within the TST framework, time perspective is not only directly related to health behaviors but also operates through health behavior motivation as a mediator, demonstrating a dual role akin to a double-edged sword. When expanding the TST framework to integrate illness perception, a chain mediation model emerged between illness perception and health behaviors, which demonstrated rigid theoretical innovation and practical application, offering a robust framework for understanding the cascading psychological processes underlying health behavior adoption and maintenance. Consequently, multifactorial intervention strategies for health behaviors that account for these interdependent influences may yield greater efficacy in facilitating behavioral change. However, due to the limitations in the research design, finite participant representativeness, and suboptimal reliability of survey instruments, this study requires further validation in large cohort samples through longitudinal research to corroborate the robustness of the findings, thereby providing a prospective framework for formulating health behavioral intervention strategies for populations with chronic conditions.

## Figures and Tables

**Table 1:** General characteristics of the participants (N = 391).

Variables	Number (%)/M ± SD
Gender	
Male	147 (37.6)
Female	244 (62.4)
Age (years)	33.12 ± 8.77
Educational level	
High school or below	8 (2.0)
Secondary school/technical school/vocational high school	7 (1.8)
Junior college	44 (11.3)
Bachelor degree	258 (66.0)
Master’s degree or above	74 (19.0)
Place of residence	
Urban	364 (93.1)
Rural	27 (6.9)
Marital status	
Unmarried	119 (30.4)
Married	267 (68.3)
Divorced	4 (1.0)
Widowed	1 (0.3)
Monthly income (RMB)	
≤3000	46 (11.8)
3001~5000	60 (15.3)
5001~8000	109 (27.9)
>8000	176 (45.0)
Employment status	
In school	47 (12.0)
Employed	332 (84.9)
Retired	3 (0.8)
Freelance work	9 (2.3)
Self-reported chronic disease	
Chronic digestive diseases	174 (44.5)
Endocrine and metabolic disorders	168 (43.0)
Cardiovascular diseases	117 (29.9)
Immune diseases	70 (17.9)
Chronic lung disease	32 (8.2)
Chronic kidney disease	12 (3.1)
Others	26 (6.4)

Note: Due to the fact that some participants reported multiple chronic diseases, the cumulative number of disease instances exceeded 391, resulting in a percentage that surpassed 100%. In addition, the percentage of some variable categories did not reach or slightly exceeded 100 due to rounding.

**Table 2:** Description of the distribution of variables.

Variables	M	SD
Past Negative	17.92	5.53
Present Impulsive	8.81	3.20
Future	21.08	2.50
Past Positive	26.06	2.85
Present Fatalistic	7.80	2.82
Illness Perception	54.15	7.64
Motivation	69.65	7.84
Health Behaviors	84.40	11.08

**Table 3:** Total and direct effects between variables.

Variables	Effect Formation	Effect Size	95% CI
Past negative → Health behaviors			
	Total	−0.407	(−0.605, −0.209)
	Direct	−0.670	(−0.858, −0.481)
Present impulsive → Health behaviors			
	Total	−0.556	(−0.912, −0.201)
	Direct	−0.997	(−1.337, −0.656)
Future → Health behaviors			
	Total	2.271	(1.884, 2.657)
	Direct	2.155	(1.789, 2.522)
Past positive → Health behaviors			
	Total	1.234	(0.873, 1.596)
	Direct	1.057	(0.708, 1.406)
Present fatalistic → Health behaviors			
	Total	−1.139	(−1.510, −0.769)
	Direct	−1.503	(−1.846, −1.160)

**Table 4:** Indirect effects between variables.

Variables	Path	Effect Size	95% CI
Past negative → Health behaviors			
	Past negative → Illness perception → Health behaviors	0.068	(0.024, 0.130)
	Past negative → Motivation → Health behaviors	0.162	(0.077, 0.256)
	Past negative → Illness perception → Motivation → Health behaviors	0.032	(0.012, 0.059)
Present impulsive → Health behaviors			
	Present impulsive → Illness perception → Health behaviors	0.069	(0.011, 0.154)
	Present impulsive → Motivation → Health behaviors	0.330	(0.176, 0.499)
	Present impulsive → Illness perception → Motivation → Health behaviors	0.042	(0.011, 0.079)
Future → Health behaviors			
	Future → Illness perception → Health behaviors	0.025	(−0.011, 0.082)
	Future → Motivation → Health behaviors	0.063	(−0.058, 0.190)
	Future → Illness perception → Motivation → Health behaviors	0.028	(0.002, 0.064)
Past positive → Health behaviors			
	Past positive → Illness perception → Health behaviors	0.030	(−0.007, 0.091)
	Past positive → Motivation → Health behaviors	0.126	(0.009, 0.259)
	Past positive → Illness perception → Motivation → Health behaviors	0.022	(0.000, 0.055)
Present fatalistic → Health behaviors			
	Present fatalistic → Illness perception → Health behaviors	0.068	(0.008, 0.158)
	Present fatalistic → Motivation → Health behaviors	0.254	(0.101, 0.426)
	Present fatalistic → Illness perception → Motivation → Health behaviors	0.042	(0.009, 0.085)

## Data Availability

The data that support the findings of this study are available upon reasonable request from the corresponding author.
